# Kielin/chordin‐like protein enhances induction of osteoblast differentiation by Bone Morphogenetic Protein‐2

**DOI:** 10.1002/2211-5463.13652

**Published:** 2023-06-01

**Authors:** Kei Nagasaki, Atsushi Yamada, Kiyohito Sasa, Ryutaro Kamijo

**Affiliations:** ^1^ Department of Biochemistry, School of Dentistry Showa University Shinagawa‐ku Japan; ^2^ Department of Orthopedic Surgery, School of Medicine Showa University Shinagawa‐ku Japan; ^3^ Department of Pharmacology, School of Medicine Showa University Shinagawa‐ku Japan

**Keywords:** BMP, C2C12 myoblasts, KCP (kielim/chordin‐like protein), osteoarthritis, osteoblasts

## Abstract

Bone morphogenetic proteins (BMPs) play a key role in embryonic differentiation for osteoblast and bone formation. Kielin/chordin‐like protein (Kcp) is known to enhance the effects of BMP signaling. Here, we present ALP activity, gene expression, and calcification data demonstrating that Kcp affects the differentiation of C2C12 myoblasts into osteoblasts. We report that the presence of Kcp enhances the ability of BMP‐2 to induce the differentiation of C2C12 myoblasts into osteoblasts. Additionally, BMP‐2‐mediated stimulation of phosphorylated Smad1/5 was apparently enhanced in the presence of Kcp. The present findings may contribute to progression toward the clinical use of BMPs for treatment of bone fracture, osteoarthritis, and other similar conditions.

## Graphical abstract

Schematic representation of interactions of BMP‐2 and Kcp with BMPR. Smad proteins are activated by phosphorylation and compose the Smad protein complex that is transferred inside of the nucleus to regulate transcription of DNA. Kcp acts to promote the phosphorylation of Smad proteins by BMP‐2 within cells via receptor activation.

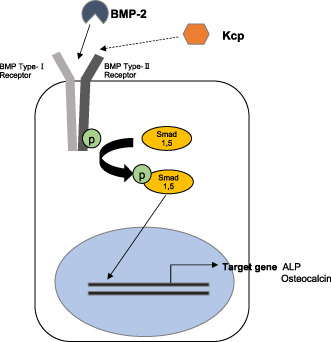

**Abbreviations**
ALP: alkaline phosphatase
BMP: bone morphogenetic protein
BMPR: bone morphogenetic protein receptor
DMEM: Dulbecco's Modified Eagle Medium
FBS: fetal bovine serum
GAPDH: glyceraldehyde 3‐phosphate
Kcp FL: kielin/chordin‐like protein full length
Kcp: kielin/chordin‐like protein
Ni‐NTA: nickel‐nitrilotriacetic acid
PCR: polymerase chain reaction
TGF: transforming growth factor

Bone morphogenetic proteins (BMPs), secreted signal transmitters that belong to the TGF‐β superfamily, are generally known to have effects causing induction of differentiation of stem cells into osteoblasts [1], with several types and various functions reported [2]. For example, BMP‐1 promotes collagenous proliferation and cartilage formation [3], while BMP‐4 promotes neural plate formation in the embryologic phase, which later develops into the central nervous system [4]. Furthermore, BMP‐6 plays an extremely important role in iron homeostasis for formation of hepcidin [5] and BMP‐15 promotes follicle formation in the ovaries [6].

Kielin/chordin‐like protein (Kcp), a large secreted protein that contains 18 cysteine‐rich domains, has become widely known for its regulatory effects on bone morphogenic proteins [7]. It is generally expressed in the kidneys and brain during all stages of the embryonic developmental process, with abundant amounts localized in renal tubules of the kidney cortex, though not within the nephrogenic zone [8]. Kcp has both suppressive and enhancing effects on some cytokines, such as enhancement of BMPs, and suppression of other cytokines such as TGF‐β or activin [7]. Although the mechanism of the effects of Kcp has not been completely revealed, it is regarded as a direct action of binding to these cytokines, due to its paracrine function [8].

The present study was conducted to investigate the sensitivity and effects of Kcp as an enhancer of BMP for induction of osteoblast differentiation utilizing C2C12 mouse cells *in vitro*. The results show that Kcp enhances the BMP effect by increasing activation of reactions of Smad protein with BMP type‐II receptors by phosphorylation.

## Materials and methods

### Reagents

Recombinant human/mouse/rat bone morphogenetic protein‐2 (BMP‐2), > 95% purified, was purchased from R&D Systems (catalog number 355‐BM; Minneapolis, MN, USA). Recombinant mouse partial length Kcp was obtained from MyBioSource, Inc. (catalog number MBS1413842; San Diego, CA, USA).

### Cell cultures

C2C12 myoblasts derived from mouse samples were used in the experiments. For cell proliferation, 15% fetal bovine serum (FBS) containing DMEM medium was used, while 2.5% FBS containing DMEM was utilized for cell differentiation.

### Determination of alkaline phosphatase activity

In order to analyze differentiation of C2C12 myoblasts into osteoblasts, alkaline phosphatase (ALP) activity was determined as a marker. Myoblasts in a 96‐well dish (1 × 10^4^cells per well) were inoculated with 15% fetal bovine serum (FBS) containing DMEM medium 1 day before treatment with reagents for cell proliferation. BMP‐2 (300 ng·mL^−1^) and Kcp (400 ng·mL^−1^) with 2.5% FBS containing DMEM were added; then, incubation was performed at 37 °C for 72 h. Following differentiation, 1% NP‐40 containing physiological saline solution was added and then subjected to destruction using an ultrasonic destructor. To determine ALP activity within the cells, an ALP activity reagent containing disodium‐P nitrophenyl phosphate hydroxylate was added and incubation performed for 15 min at 37 °C, and then, absorbance at 405 nm was measured.

### Nickel pull‐down assay of ALP activity with Kcp full‐length plasmid

C2C12 myoblasts in 6‐well dishes (1 × 10^5^ cells per well) containing DMEM with 2.5% FBS were inoculated with Kcp full‐length (Kcp FL) plasmid, constructed by VectorBuilder Japan and termed pPB[Exp]‐Puro‐EF1A > lgκ‐sig/mKcp[NM‐00102998.4]/Myc/6xHis (VB220208‐1023cxt), and Lipofectamine 3000 (Thermo Fisher Scientific); then, incubation was performed at 37 °C for 72 h. Using 2 mL of supernatant from a dish with cell lysate containing histidine (His) tagged with full‐length Kcp, a nickel pull‐down assay was conducted. Ni‐NTA agarose beads were prepared with a high affinity for His‐tagged proteins and used with His‐tagged full‐length Kcp to bind to the nickel‐charged resin, after which the resin was washed thoroughly to remove any unbound or nonspecifically bound proteins. Finally, the His‐tagged protein was eluted from the resin using a buffer containing a high concentration of imidazole, which competes with the His residues for binding to nickel ions. A sample containing 0.5 mL of the eluted Kcp FL was utilized as a reagent for determining ALP activity performed with partial length Kcp. Myoblasts in a 96‐well dish (1 × 10^4^ cells per well) were inoculated with 15% fetal bovine serum (FBS) containing DMEM medium 1 day before treatment with reagents for cell proliferation. Finally, DMEM containing 2.5% FBS with BMP‐2 and 10% of the Kcp FL from the nickel pull‐down assay were added, and incubation was performed at 37 °C for 72 h.

### ALP activity staining

After culturing with reagents for BMP‐2 and Kcp, cells were treated with 10% formalin for 20 min at room temperature, then an acetone/ethanol solution was added, and incubation continued for 1 min at room temperature. For ALP activity staining, cells were treated with an ALP staining solution containing Naphthol AS‐mX and Fast blue BB salt and then semi‐dissolved with N‐N diethyl formalin for 15 min at 37 °C. Purple staining in the cells was considered to indicate the portion possessing ALP activity.

### Alizarin red staining

To determine the amounts that showed differentiation into osteoblasts, the degree of osteoblast calcification was determined using Alizarin red staining and its absorbance. In the same manner noted above, C2C12 myoblasts in a 96‐well dish (1 × 10^4^ cells per well) were inoculated with 15% FBS containing DMEM medium 1 day before treatment with reagents for cell proliferation. BMP‐2 (100 ng·mL^−1^) and Kcp (500 ng·mL^−1^) were added with 2.5% FBS containing DMEM, and then, incubation was performed at 37 °C for 72 h. Culturing was then continued for 11 more days with differentiation medium containing ascorbic acid (50 μL·mL^−1^), β‐glycerophosphate (10 mm), and dexamethasone (10 nm), which was changed every 3 days. For staining, cells were treated with 95% ethanol for 20 min at room temperature; then, a calcification staining mixture containing 1% Alizarin red S and ammonium solutions was added for 5 min at room temperature. Parts with dark reddish staining were considered to indicate osteoblast calcification. Consequently, after drying the stained cells, 10% cetylpyridinium chloride hydrate was applied to dissolve them and absorbance at 570 nm was measured.

### ALP and osteocalcin gene expressions

Quantitative evaluation of osteoblast mRNA expression in C2C12 cells in the presence or absence of BMP‐2 and Kcp was performed. C2C12 myoblasts in a 6‐well dish (1 × 10^5^cells per well) were inoculated with BMP‐2 (100 ng·mL^−1^) or Kcp (500 ng·mL^−1^) with 2.5% FBS containing DMEM and then incubated at 37 °C for 72 h. Expression of marker genes, such as ALP and osteocalcin, was evaluated for determining osteoblast differentiation. Total RNA was extracted from C2C12 cells using TRIzol reagent (Life Technologies, Carlsbad, CA, USA). After inducing a reverse transcription reaction with 5× RT Master mix (catalog number FSQ‐201; TOYOBO, Kita‐ku, Osaka, Japan), gene expressions were quantified using real‐time PCR with THUNDERBIRD® qPCR Mix (catalog number QPS‐101, TOYOBO) for *ALP* and with glyceraldehyde 3‐phosphate (GAPDH) from a TaqMan™ Gene Expression Assay Kit (Thermo Fisher Scientific) for *osteocalcin*, and then, the levels of expression were normalized based on the value for GAPDH.

### Western blotting analysis

C2C12 myoblasts in a 6‐well dish (1 × 10^5^cells per well) were inoculated with BMP‐2 (100 ng·mL^−1^) and Kcp (500 ng·mL^−1^) with 2.5% FBS containing DMEM; then, incubation was performed for 37 °C for 72 h. Degraded cell lysates were collected using sample buffer solution without a reducing reagent (6×) for SDS/PAGE (NACALAI TESQUE, Inc, Kyoto, Japan); then, the samples were transferred to an Immobilon‐P membrane (Millipore Corp., Bedford, MA, USA). Antiphosphorylated Smad1/5, anti‐Smad1, anti‐Smad5, and anti‐actin primary antibodies (Cell Signaling Technology Japan, Tokyo, Japan), and the secondary antibodies anti‐Rabbit IgG and HRP‐Linked Whole Ab Donkey (Cytiva Global Life Science Technologies Japan, Tokyo, Japan) were used to detect total Smad1, Smad5, pSmad1/5, and actin. The Quantity One software package, developed by Bio‐Rad Laboratories (Hercules, CA, USA) for analysis of blot images, was used for objective determinations of intensity and band area (Fig. 4B). This package allows users to analyze, quantify, and compare protein and nucleic acid samples in a variety of blot formats, including western blots. Quantity One supports a range of image formats, including TIFF and JPEG for image acquisition. The images can be edited manually to enclose lanes and bands, such as enclosure with a square. The software then calculates the intensity and area of the bands, and automatically performs volume analysis.

## Results and Discussion

### ALP activity measurement, and quantitative analysis of ALP and osteocalcin gene expression for differentiation into osteoblasts

**Fig. 1:**
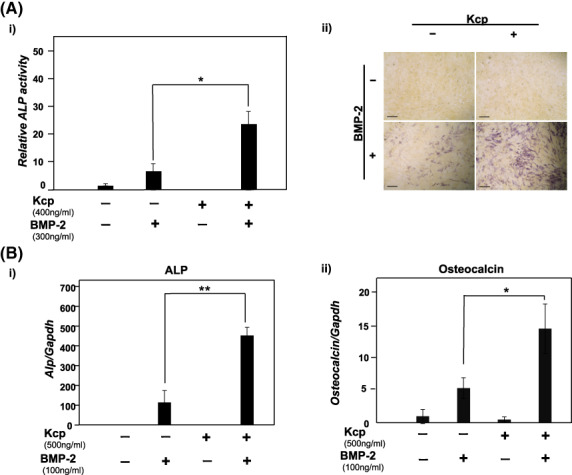
Determination of ALP activity, and gene expressions of ALP and Osteocalcin. Kcp accelerates the effect of BMP‐2 to induce differentiation into osteoblasts. (A(i)) Measurement of ALP activity. (A(ii)) ALP activity staining. BMP‐2 (300 ng·mL^−1^) and Kcp (400 ng·mL^−1^) were added to C2C12 cells derived from mouse myoblasts. After 72 h of incubation at 37 °C, cells were subjected to destruction with an ultrasonic destructor in order to measure ALP activity within the cells by determining absorbance. Relative values for each group were calculated based on a control group value of 1. (B) Gene expression of ALP and Osteocalcin as marker genes for osteoblast differentiation. C2C12 cells derived from mouse myoblasts were used with BMP‐2 and Kcp added. The level of ALP gene expression was analyzed using a quantitative PCR method with a TaqMan probe. ALP expression level was corrected using GAPDH expression level as the intrinsic control. The relative expression level of each group was calculated using an ALP/GAPDH value of 1 for the group without BMP‐2 and Kcp added (control). Results are shown as the mean ± SD of three samples. **P < 0.01 and *P < 0.05, Student's *t*‐test. Scale bars = 200 μm.

Results showing promotion of differentiation of C2C12 myoblasts into osteoblasts with BMP‐2 and Kcp are presented in Fig. 1. For this analysis, increased ALP activity was considered to indicate increased differentiation into osteoblasts. With stable concentrations of BMP‐2 and Kcp, the activity of ALP was apparently increased in the presence of BMP‐2, while addition of Kcp enhanced that increase induced by BMP‐2 (Fig. 1A(i)). ALP activity staining was also performed to visualize the increased activity, with purple staining indicating osteoblasts. Without reagents or in the presence of Kcp alone, no color change was observed, whereas under the condition with both BMP‐2 and Kcp, several of the cells changed to purple (Fig. 1A(ii)). Additionally, quantitative mRNA expression of ALP and osteocalcin genes were analyzed by normalizing their values with the value of GAPDH. The amounts of ALP and osteocalcin genes were obviously increased in the presence of BMP‐2 with Kcp as compared to BMP‐2 alone (Fig. 1B). As a result, the induction ability of BMP‐2 for differentiation of C2C12 myoblasts into osteoblasts was enhanced in the presence of Kcp. Furthermore, these findings showed that Kcp alone does not have a function to directly enhance the induction effect, as BMP is required to react to increased cell signaling. The activity of Kcp FL was also determined. Kcp FL is generated by Kcp FL plasmid vector transfection and eluted with an Ni pull‐down assay. Using the same methods noted above, experiments for measuring ALP activity were performed and the results indicated that Kcp FL also has an enhanced effect to block induction of BMP‐2 into osteoblasts (Fig. S1).

### Effects of BMP‐2 on differentiation into osteoblasts with different concentrations of Kcp

**Fig. 2:**
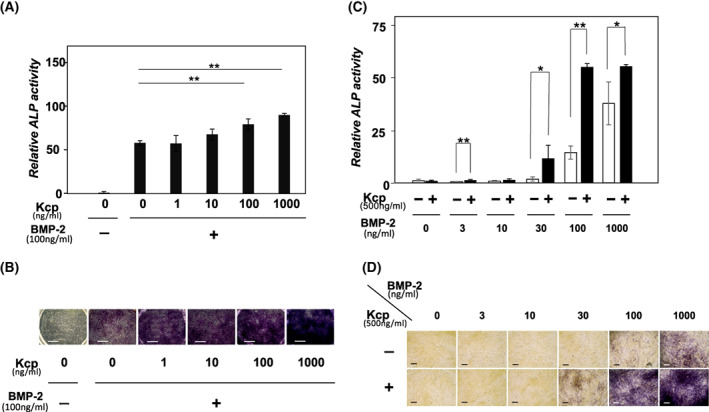
Determination of ALP activity with various concentrations of reagents. (A) C2C12 cells with a stable concentration of BMP‐2 (300 ng·mL^−1^) and various concentrations of Kcp (1–1000 ng·mL^−1^). (B) ALP activity staining. Scale bars = 1000 μm. (C) C2C12 cells with a stable concentration of Kcp and various concentrations of BMP‐2 (3–1000 ng·mL^−1^). After 72 h of incubation at 37 °C, cells were subjected to destruction with an ultrasonic destructor; then, ALP activity within the cells was determined by measuring absorbance. (D) ALP activity staining. Scale bars = 200 μm. Results are shown as the mean ± SD of three samples. **P < 0.01 and *P < 0.05, Student's *t*‐test.

ALP activity was measured after altering the concentrations of BMP‐2 and Kcp. Analysis of the increased rate of ALP activity with a stable concentration of BMP‐2 (100 ng·mL^−1^) and various concentrations of Kcp (0–1,000 ng·mL^−1^) noted statistically significant values with a Kcp concentration of 100 ng·mL^−1^ (Fig. 2A,B), indicating that an extremely low concentration of Kcp has no effect on ALP activity. Moreover, according to the results shown by a graph, ALP activity did not increase in a dose‐dependent manner as seen with Kcp. Furthermore, using a stable concentration of Kcp (500 ng·mL^−1^), a suitable concentration of BMP‐2 for inducing a significant increase in ALP was examined, with the results indicating that > 100 ng·mL^−1^ of BMP‐2 had an adequate effect to increase the activity of ALP (Fig. 2C,D).

### Enhancement of BMP‐2‐induced calcification in osteoblasts by Kcp

**Fig. 3:**
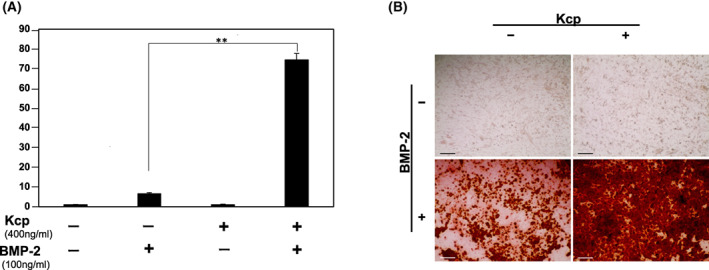
Alizarin red staining for analyzing calcification of osteoblasts. C2C12 cells derived from mouse myoblasts were used to examine the effects of BMP‐2 and Kcp. The time course for differentiation and calcification of the osteoblasts was 14 days. Differentiation medium containing ascorbic acid, β‐glycerophosphate (10 mm), and dexamethasone was used, with that changed every 3 days. (A) Determination of calcification based on absorbance of Alizarin red. (B) Alizarin red staining. Dark red areas indicate osteoblast calcification. Results are shown as the mean ± SD of three samples. **P < 0.01, Student's *t*‐test. Scale bars = 200 μm.

To examine calcification of osteoblasts, use of Alizarin red staining was effective to determine differentiation into osteoblasts. First, C2C12 cells were incubated with BMP‐2 and Kcp for 3 days, and then, the conditions were altered to differentiation medium lacking BMP‐2 or Kcp, which was changed every 3 days. On Day 14, there was an obvious increase in number of cells stained with Alizarin red when incubated in the presence of both BMP‐2 and Kcp, with similar results noted when in the presence of BMP‐2 alone (Fig. 3A,B). In contrast, nearly no calcification was observed in the control cells or when incubated with only Kcp.

### Kcp shows indirect enhancement of Smad protein activation

**Fig. 4:**
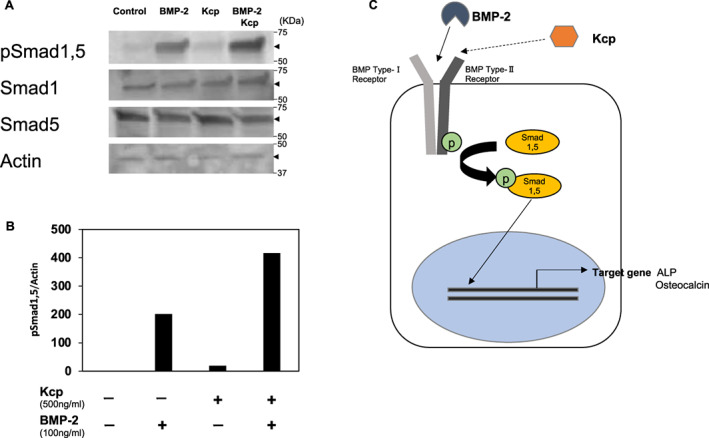
Western blotting analysis to detect activation of Smad with phosphorylation. (A) Band showing phosphorylated Smad 1/5 protein with application of both BMP‐2 and Kcp indicates an apparently increase as compared to without Kcp application. There were no differences regarding the protein amounts of Smad1, Smad5, and actin among the groups. (B) Graph showing relative values of adjusted volume of protein bands for pSmad1/5. The adjusted volume of pSmad1/5 was determined based on the adjusted volume of actin. Relative values for each group were determined using a value of 1 for the control group. (C) Cell signaling pathway showing activity of BMP‐2 and Kcp to phosphorylate Smad.

TGF‐β signals are mainly transmitted by transcription factor proteins located in cells with Smad. Smad proteins are activated by phosphorylation and compose the Smad protein complex that is transferred inside of the nucleus to regulate transcription DNA [9]. To examine the level of activity and means of increasing the effect of BMP‐2 by Kcp, western blotting analysis was performed to detect the amount of activated Smad proteins in each sample. Accumulation of phosphorylated Smad1/5 by BMP‐2 stimulation was apparently enhanced in its presence (Fig. 4A,B), suggesting that Kcp acts to promote the phosphorylation of Smad proteins within cells via receptor activation (Fig. 4C).

### Enhancing effect of Kcp on BMP‐2 and its effectiveness

Previous studies have shown the existence of antagonists that bind to BMP and inhibit its function. These are mainly classified into four types, that is, Chordin, Noggin, DAN family, and Folistatin, and indicated to inhibit the function of BMP with a negative feedback system [10]. In contrast to those antagonists, a few reports have noted factors that enhance the effects of BMP. As mentioned above, Kcp, which possesses abundant cysteine residues, has been detected in an embryonic renal mesenchyme cDNA library [8], and reported to contain 18 cysteine‐rich coding repeats and a von Willebrand factor D domain [11].

Generally, induction of bone formation by mammalian cells requires a large amount of BMP. Based on the present findings showing the enhancing effect of Kcp on BMP‐2, it is speculated that this BMP agonist diminishes the amount of BMPs available for regeneration and formation of bone. Heparin, composed of sulfated polysaccharides and regarded as another BMP agonist, similarly enhances BMPs to inhibit their transformation into myogenic cells, and induction of osteoblast and bone formation [12]. A previous study found that 5 μg·mL^−1^ of heparin had an effect on differentiation of C2C12 cells into osteoblasts with 100 ng·mL^−1^ of BMP‐2 [12]. In the present experiments, in comparison with the effect of heparin, 100 ng·mL^−1^ of Kcp produced an adequate effect for 100 ng·mL^−1^ of BMP‐2, with a significant difference (Fig. 2A,B). Furthermore, use of 500 ng·mL^−1^ of Kcp provided nearly the maximum effect with 100 ng·mL^−1^ of BMP‐2 on C2C12 cells for osteoblast differentiation (Fig. 2C,D). Regarding the effectiveness of the enhancing effect on BMP‐2, it is suggested that Kcp is superior to heparin for osteoblastic differentiation.

It has been shown that Kcp can bind directly to BMP‐7 and promote binding to BMPR‐1A, leading to positive regulation of BMP signaling [8]. That study also found that Kcp binds directly to BMP‐7 and enhances BMP‐7‐mediated phosphorylation of Smad1/5/8, a downstream signaling event of BMP signaling. Based on the present findings, it is considered that the same occurs with respect to the interaction of BMP‐2 and Kcp. Additional studies will be needed to fully elucidate the molecular mechanisms underlying this interaction.

In conclusion, the present results indicate that as compared to its presence alone, a low amount of BMP‐2 with Kcp has a definite effect on enhancing ALP activity to induce differentiation of C2C12 cells into osteoblasts.

## Conflict of interest

The authors declare no conflict of interest.

### Peer Review

The peer review history for this article is available at https://www.webofscience.com/api/gateway/wos/peer‐review/10.1002/2211‐5463.13652.

## Author contributions

KN, AY, and RK designed the experiments. KN and KS performed corresponding experiments. KN wrote the paper.

## Supporting information


**Fig. S1.** Determination of ALP activity and Kcp FL staining.

## Data Availability

The datasets analyzed during the current study are available from the corresponding author on reasonable request.

## References

[1] Chen G , Deng C and Li YP (2012) TGF‐beta and BMP signaling in osteoblast differentiation and bone formation. Int J Biol Sci 8, 272–288. 22298955 10.7150/ijbs.2929 PMC3269610

[2] Chen Y , Ma B , Wang X , Zha X , Sheng C , Yang P and Qu S (2021) Potential functions of the BMP family in bone, obesity, and glucose metabolism. J Diabetes Res 2021, 6707464. 34258293 10.1155/2021/6707464 PMC8249130

[3] Kessler E , Takahara K , Biniaminov L , Brusel M and Greenspan DS (1996) Bone morphogenetic protein‐1: the type I procollagen C‐proteinase. Science 271, 360–362. 8553073 10.1126/science.271.5247.360

[4] Moon BS , Yoon JY , Kim MY , Lee SH , Choi T and Choi KY (2009) Bone morphogenetic protein 4 stimulates neuronal differentiation of neuronal stem cells through the ERK pathway. Exp Mol Med 41, 116–125. 19287192 10.3858/emm.2009.41.2.014 PMC2679327

[5] Denardo A , Elli S , Federici S , Asperti M , Gryzik M , Ruzzenenti P , Carmona F , Bergese P , Naggi A , Arosio P *et al*. (2021) BMP6 binding to heparin and heparan sulfate is mediated by N‐terminal and C‐terminal clustered basic residues. Biochim Biophys Acta Gen Subj 1865, 129799. 33232799 10.1016/j.bbagen.2020.129799

[6] Persani L , Rossetti R , Di Pasquale E , Cacciatore C and Fabre S (2014) The fundamental role of bone morphogenetic protein 15 in ovarian function and its involvement in female fertility disorders. Hum Reprod Update 20, 869–883. 24980253 10.1093/humupd/dmu036

[7] Lin J , Patel SR , Wang M and Dressler GR (2006) The cysteine‐rich domain protein KCP is a suppressor of transforming growth factor beta/activin signaling in renal epithelia. Mol Cell Biol 26, 4577–4585. 16738323 10.1128/MCB.02127-05 PMC1489124

[8] Lin J , Patel SR , Cheng X , Cho EA , Levitan I , Ullenbruch M , Phan SH , Park JM and Dressler GR (2005) Kielin/chordin‐like protein, a novel enhancer of BMP signaling, attenuates renal fibrotic disease. Nat Med 11, 387–393. 15793581 10.1038/nm1217

[9] Blank U and Karlsson S (2011) The role of Smad signaling in hematopoiesis and translational hematology. Leukemia 25, 1379–1388. 21566654 10.1038/leu.2011.95

[10] Glister C , Regan SL , Samir M and Knight P (2018) Gremlin, noggin, chordin and follistatin differentially modulate BMP induced suppression of androgen secretion by bovine ovarian theca cells. J Mol Endocrinol. 10.1530/JME-18-0198 30400042

[11] Garcia Abreu J , Coffinier C , Larrain J , Oelgeschlager M and De Robertis EM (2002) Chordin‐like CR domains and the regulation of evolutionarily conserved extracellular signaling systems. Gene 287, 39–47. 11992721 10.1016/s0378-1119(01)00827-7

[12] Zhao B , Katagiri T , Toyoda H , Takada T , Yanai T , Fukuda T , Chung UI , Koike T , Takaoka K and Kamijo R (2006) Heparin potentiates the in vivo ectopic bone formation induced by bone morphogenetic protein‐2. J Biol Chem 281, 23246–23253. 16754660 10.1074/jbc.M511039200

