# Supplementary material for: Kielin/chordin‐like protein enhances induction of osteoblast differentiation by Bone Morphogenetic Protein‐2
Source: FEBS Open Bio. 2023 Jun 1;13(7):1357–64. doi: 10.1002/2211-5463.13652 (PMC10315795; doi:10.1002/2211-5463.13652)
Supplement: Supplementary file 1 — Fig. S1. Determination of ALP activity and Kcp FL staining. [file FEB4-13-1357-s001.pdf]

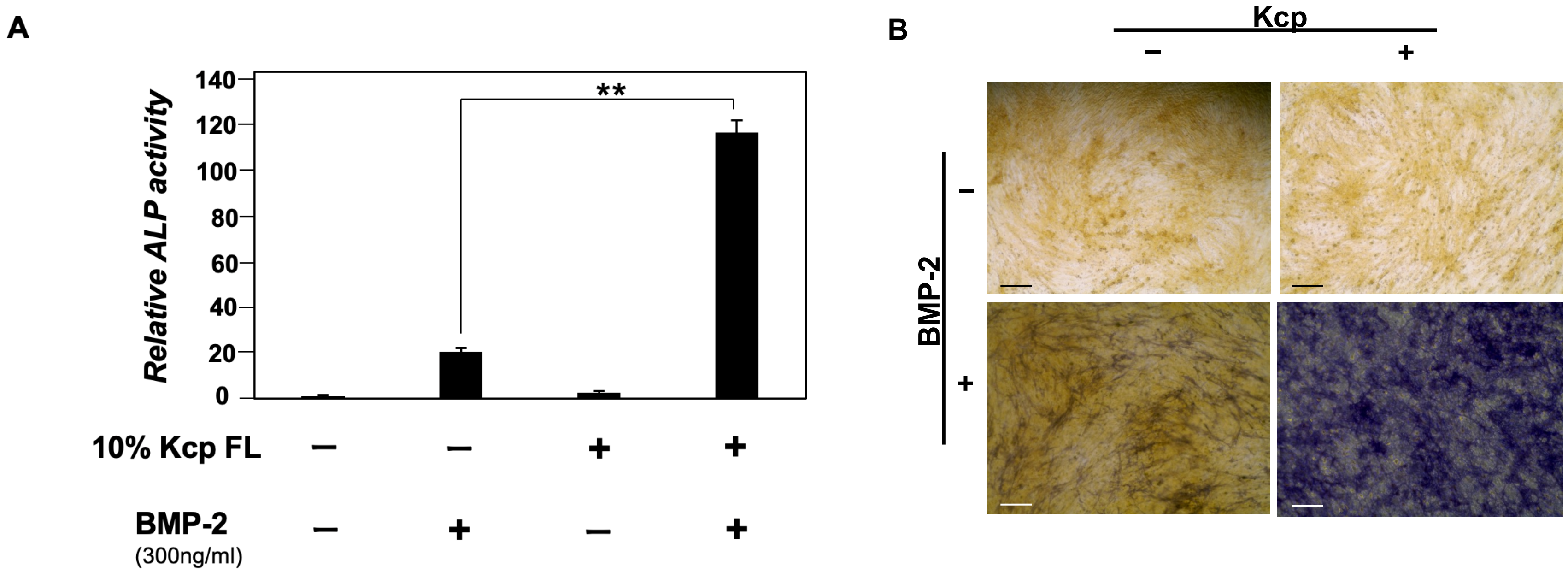

Suppl. Fig. 1. Determination of ALP activity and Kcp FL staining.

Kcp FL was found to accelerate the effect of BMP-2 to induce differentiation into osteoblasts. (A) Determination of ALP activity. (B) ALP activity staining. BMP-2 (300 ng/ml) and 10% Kcp FL were added to C2C12 cells derived from mouse myoblasts. Using the same methods for the results noted in A, after 72 hours of incubation at 37˚C, cells were subjected to destruction with an ultrasonic destructor, then ALP activity within the cells was examined by determining absorbance. Relative values for each group were calculated based on a control group value of 1. Results are shown as the mean±SD of three samples. **P<0.01, Student's t test. Scale bars=200μm.
